# Supraspinal-selective TRPV1 desensitization induced by intracerebroventricular treatment with resiniferatoxin

**DOI:** 10.1038/s41598-017-12717-5

**Published:** 2017-09-29

**Authors:** Akihiro Fukushima, Kizuku Mamada, Aki Iimura, Hideki Ono

**Affiliations:** 1 Laboratory of Clinical Pharmacy and Pharmacology, Faculty of Pharmacy, Musashino University, 1-1-20 Shinmachi, Nishitokyo-shi, Tokyo, 202-8585 Japan; 2 Research Institute of Pharmaceutical Sciences, Musashino University, 1-1-20 Shinmachi, Nishitokyo-shi, Tokyo, 202-8585 Japan

## Abstract

The transient receptor potential vanilloid type 1 (TRPV1) is a thermosensitive cation channel that triggers heat pain in the periphery. Long-term desensitization of TRPV1, which can be induced by excess amounts of agonists, has been a method for investigating the physiological relevance of TRPV1-containing neuronal circuits, and desensitization induced by various routes of administration, including systemic, intrathecal and intraganglionic, has been demonstrated in rodents. In the present study, we examined the effect of intracerebroventricular (i.c.v.) treatment with an ultrapotent TRPV1 agonist, resiniferatoxin (RTX), on nociception and the analgesic effect of acetaminophen, which is known to mediate the activation of central TRPV1. I.c.v. administration of RTX a week before the test did not affect the licking/biting response to intraplantar injection of RTX (RTX test), suggesting that such i.c.v. treatment spares the function of TRPV1 at the hindpaw. Mice that had been i.c.v.-administered RTX also exhibited normal nociceptive responses in the formalin test and the tail pressure test, but acetaminophen failed to induce analgesia in those mice in any of the tests. These results suggest that i.c.v. administration of RTX leads to brain-selective TRPV1 desensitization in mice.

## Introduction

The transient receptor potential vanilloid type 1 (TRPV1) is a nonselective cation channel that acts as a polymodal sensor for several types of stimuli (capsaicin, heat, low pH and osmolality)^1^. Among primary sensory neurons, TRPV1 is mainly expressed in nonmyelinated peptidergic C-fibers and a portion of Aδ fibers^2^. In the spinal cord, TRPV1 is present on the central terminals of primary afferents and postsynaptic second-order neurons in the dorsal horn^3^. These peripheral and spinal TRPV1-expressing neurons are thought to transmit noxious heat and mechanical stimuli to the brain^4,5^. It is considered that TRPV1 is also expressed in several supraspinal CNS regions^6^, although the precise expression pattern is controversial^7^. It has been suggested that supraspinal TRPV1 activation modulates pain sensation; microinjection of capsaicin into the ventrolateral periaqueductal gray increases the threshold of thermal pain sensitivity^8^. TRPV1 has also been suggested to play some functional roles in other brain regions. Thus CNS-selective TRPV1-deficient mice would be a better model for understanding the function of TRPV1 in the CNS.

It is known that an administration of excess TRPV1 agonist leads to cytotoxic calcium overload and cell death of TRPV1-positive neurons^9^. Animals treated in this way show long-lasting desensitization to TRPV1 agonists^10,11^, and in desensitized rats it has been demonstrated that capsaicin-sensitive structures in the brain play a key role in the analgesic action of morphine^12,13^. The site of desensitization and its extent depend on the administration route and dose of agonist; for example, high-dose (0.3 mg/kg) subcutaneous (s.c.) administration of resiniferatoxin (RTX), an ultrapotent TRPV1 agonist^14^, induces systemic desensitization: loss of the eye-wiping response to corneal capsaicin application and RTX binding in the dorsal root ganglia of rats^10^. A lower dose of intraperitoneal RTX (0.02 mg/kg) desensitizes only the peritoneal cavity without affecting paw thermal sensitivity and the eye-wiping response^15^. Intrathecal (i.t.) injection of RTX or capsaicin eliminates TRPV1-expressing central nerve terminals in the spinal dorsal horn^16,17^. Microinjection of RTX within the trigeminal ganglia can induce selective loss of ipsilateral TRPV1-immunopositive neurons^18^. Although this chemical ablation approach has been used to investigate the function of TRPV1, preceding studies have focused on TRPV1-positive primary afferents. To our knowledge, supraspinal-selective desensitization has not yet been demonstrated.

Acetaminophen, a widely-used analgesic drug, is thought to exert its analgesic effect through activation of the descending serotonergic system^19,20^. Acetaminophen is deacetylated in the liver, and the deacetylated metabolite 4-aminophenol is converted to the TRPV1 agonist N-(4-hydroxyphenyl) arachidonamide (AM404) through conjugation with arachidonic acid in the brain. It has been reported that acetaminophen-induced analgesia is inhibited by intracerebroventricular (i.c.v.) administration of capsazepine, a competitive TRPV1 antagonist, and TRPV1 gene knockout, suggesting that acetaminophen induces analgesia through activation of central TRPV1^21^. On the basis of these reports, we hypothesized that the analgesic effect of acetaminophen would be inhibited in RTX-treated mice if i.c.v.-administered RTX selectively desensitizes TRPV1 at supraspinal sites.

In the present study using mice, we assessed the effect of i.c.v. pretreatment with RTX on nociception and the effect of acetaminophen.

## Results

### RTX test: nociceptive behavior evoked by direct activation of TRPV1 at the hindpaw

**Figure 1:**
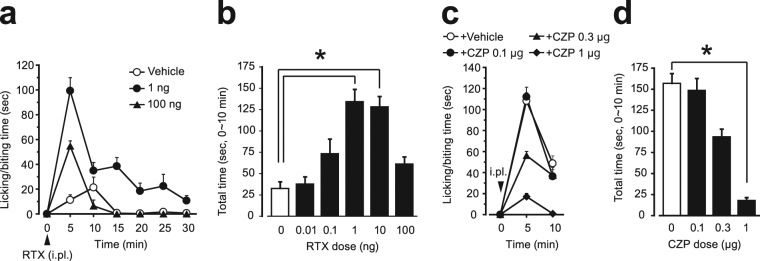
Nociceptive behavior evoked by i.pl. injection of resiniferatoxin (RTX test) and the inhibitory effect of capsazepine. (**a**) Time course of licking/biting behavior evoked by i.pl. injection of RTX (0.01-100  ng). RTX was injected at time zero (arrowhead). For clarity, the results for 0.01, 0.1 and 10 ng RTX are not shown. (**b**) Time spent licking/biting during the first 10 minutes of the test. (**c**, **d**) Time course of licking/biting behavior evoked by i.pl. injection of RTX (1 ng) and time spent licking/biting during the first 10 minutes of the test. Capsazepine was co-injected with RTX at time zero (arrowhead in **c**). All data represent the mean ( ± SEM) result from six and five mice in (**a**, **b**) and (**c**, **d**) respectively. Non-parametric multiple comparisons with Bonferroni correction following the Kruskal-Wallis test were used [(**b**), five comparisons in six groups; (**d**), three comparisons in four groups; *P < 0.05 vs. Vehicle group]. CZP, capsazepine; i.pl., intraplantar injection.

We first assessed the nociceptive behavior evoked by intraplantar injection of RTX. Injected mice showed robust nociceptive behavior such as licking/biting, flinching, shaking and lifting the injected paw. The licking/biting behavior was remarkable in the first 10 min, and then became gradually reduced (Fig. 1a). There was no following phase that can be observed in the formalin test. At lower doses (0.01, 0.1 and 1 ng), intraplantar RTX increased the licking/biting behavior dose-dependently, and the most effective dose was 1 ng. In turn, the licking/biting behavior was decreased when the RTX dose was increased to 100 ng (Fig. 1b). Co-injection of capsazepine (0.1-1 μg) with RTX (1 ng) reduced the duration of the nociceptive behavior in a dose-dependent manner (Fig. 1c,d). Based on these results, we selected 1 ng RTX in a 20 μL injection volume and assessed the total licking/biting time during the first 10 min in the following experiments.

### Pretreatment with intracerebroventricular resiniferatoxin preserves TRPV1 at the hindpaw

**Figure 2:**
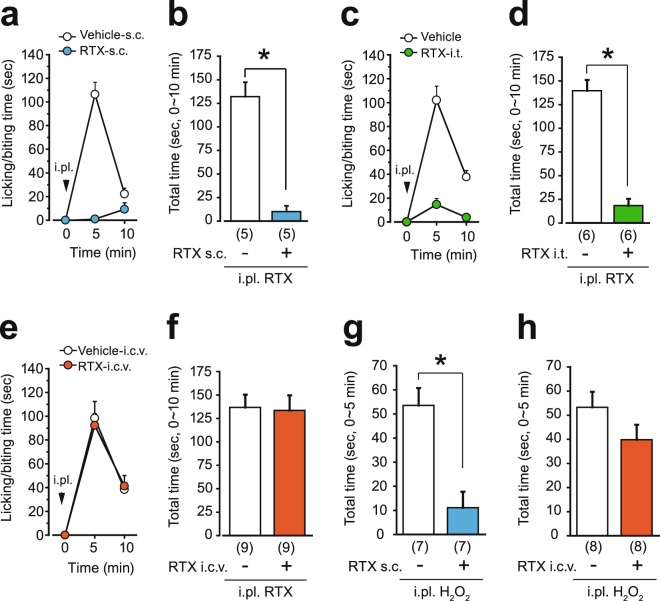
I.c.v. administration of RTX spares the function of TRPV1 at the hindpaw. Mice were pretreated with RTX one week before the tests [(**a**, **b**, **g**) s.c. pretreatment; (**c**, **d**) i.t. pretreatment; (**e**, **f**, **h**) i.c.v. pretreatment]. Time course of licking/biting behavior evoked by i.pl. injection of RTX (1 ng to the hindpaw, arrowheads in **a**, **c**, **e**) and total time spent licking/biting during the first 10 minutes of the tests. (**g**, **h**) Time spent licking/biting behavior in response to i.pl. injection of 0.3% H_2_O_2_. All data represent the mean ± SEM. The number of mice in each group is shown in parentheses. The Mann-Whitney U-test was used (*P < 0.05). i.pl., intraplantar injection.

We next examined the nociceptive behavior in the RTX test of mice that had been pretreated subcutaneously (0.2 mg/kg), intrathecally (100 ng) and intracerebroventricularly (100 ng) with RTX. If the effect of i.c.v.-RTX was limited to the supraspinal region, then the RTX-i.c.v.-pretreated mice would show normal nociceptive behavior in the RTX test. Mice were pretreated with RTX a week before the tests. In the s.c.-pretreated and i.t.-pretreated mice, RTX-evoked licking/biting behavior was abolished almost completely (Fig. 2a-d). On the other hand, the i.c.v.-pretreated mice exhibited licking/biting behaviors comparable to that of vehicle-pretreated mice (Fig. 2e,f). The appearance and body weight of i.c.v.-pretreated mice were normal (Vehicle group, 29.9 ± 1.1 g, n = 9; RTX group, 29.8 ± 1.4 g, n = 9). Spontaneous activity of the i.c.v.-pretreated mice in a novel environment was comparable to that of vehicle-pretreated mice (Supplementary Fig. S1a), ruling out any possible change in the expression of nociceptive behavior due to hyper- or hypo-activity. The brain monoamine content (serotonin, noradrenaline and dopamine) in the i.c.v.-pretreated mice was also unchanged (Supplementary Fig. S1b-d).

It has been reported that the transient receptor potential ankyrin type 1 (TRPA1) is co-expressed with TRPV1 in primary afferents^22^, and that systemic treatment with RTX also removes TRPA1^23^. Therefore, we next assessed hydrogen peroxide-evoked, TRPA1-dependent nociceptive behavior in RTX-pretreated mice^24–26^. As was the case for the RTX test, i.c.v.-pretreated mice showed licking/biting behavior after intraplantar injection of 0.3% H_2_O_2_ solution, whereas s.c.-pretreated mice did not (Fig. 2g,h).

**Figure 3:**
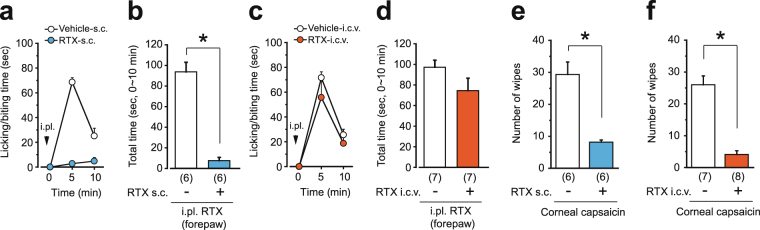
I.c.v. administration of RTX spares the function of TRPV1 at the forepaw but not at the cornea. Mice were pretreated with RTX one week before the tests [(**a**, **b**, **e**) s.c. pretreatment; (**c**, **d**, **f**) i.c.v. pretreatment]. The time course of licking/biting behavior evoked by i.pl. injection of RTX (1 ng to the forepaw, arrowheads in **a**, **c**) and the total time spent licking/biting during the first 10 minutes of the tests. (**e**, **f**) The number of eye-wiping episodes evoked over a 3-min period by instillation of capsaicin to the cornea. All data represent the mean ± SEM. The number of mice in each group is shown in parentheses. The Mann-Whitney U-test was used (*P < 0.05). i.pl., intraplantar injection.

It has been shown that intracisternal capsaicin administration reduces the eye-wiping response to corneal application of capsaicin^27^ and orofacial heat pain^28^. To assess the effect of i.c.v.-injected RTX on nociceptors in the more rostral part of the body, we also examined the change in responsiveness to TRPV1 agonists at the forepaw and the eye of RTX-pretreated mice. As was the case for the hindpaw, intraplantar injection of RTX (1 ng) to the forepaw also evoked licking/biting behavior, and this behavior was abolished in RTX-s.c.-pretreated mice (Fig. 3a,b). On the other hand, RTX-i.c.v.-pretreated mice responded to forepaw injection of RTX (Fig. 3c,d). Neither s.c.- nor i.c.v.-pretreated mice exhibited the eye-wiping response to instillation of 0.1% capsaicin solution (Fig. 3e,f).

### RTX-i.c.v. pretreatment does not alter nociceptive responses but attenuates the effect of acetaminophen

**Figure 4:**
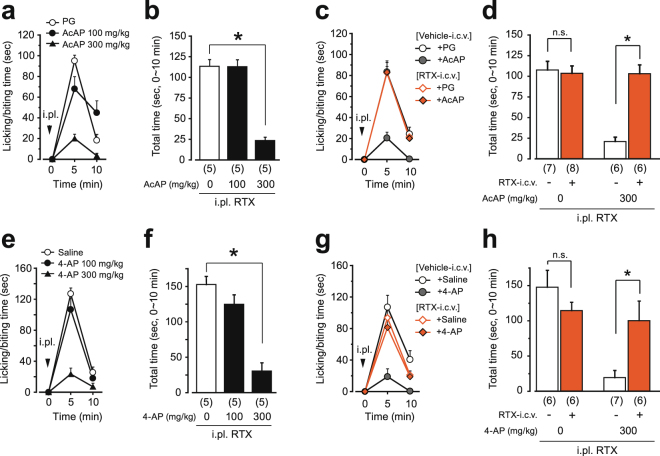
Acetaminophen and 4-aminophenol reduce nociceptive behavior evoked by i.pl. injection of RTX in naive mice but not in mice i.c.v.-administered RTX. Time course of licking/biting behavior evoked by i.pl. injection of RTX (1 ng to the hindpaw, arrowheads in **a**, **c**, **e**, **g**) and total time spent licking/biting during the first 10 minutes of the tests are shown. Naive, untreated mice were used in **a**, **b**, **e** and **f**. In **c**, **d**, **g** and **h**, mice were pretreated with RTX (100 ng, i.c.v.) or vehicle one week before the tests. Acetaminophen and 4-aminophenol hydrochloride were i.p.-administered 20 min before i.pl. injection. All data represent the mean ± SEM. The number of mice in each group is shown in parentheses. Non-parametric multiple comparisons with Bonferroni correction following the Kruskal-Wallis test were used for **b** and **f** (two comparisons in three groups, *P < 0.05 vs. Vehicle group). The Mann-Whitney U-test was used for **d** and **h** (*P < 0.05 vs. Vehicle-i.c.v. group). PG, propyleneglycol; AcAP, acetaminophen; 4-AP; 4-aminophenol hydrochloride; n.s., not significant; i.pl., intraplantar injection.

We next examined whether acetaminophen and its metabolite 4-aminophenol reduced the nociceptive behavior of untreated (naive) mice in the RTX test at the hindpaw. Both acetaminophen and 4-aminophenol hydrochloride reduced the licking/biting behavior at a dose of 300 mg/kg (Fig. 4a,b,e,f). Neither indomethacin nor diclofenac sodium salt reduced the licking/biting behavior at a dose of 30 mg/kg (Supplementary Fig. S2), although this dose was sufficient to inhibit inflammatory pain in the formalin test^29,30^.

**Figure 5:**
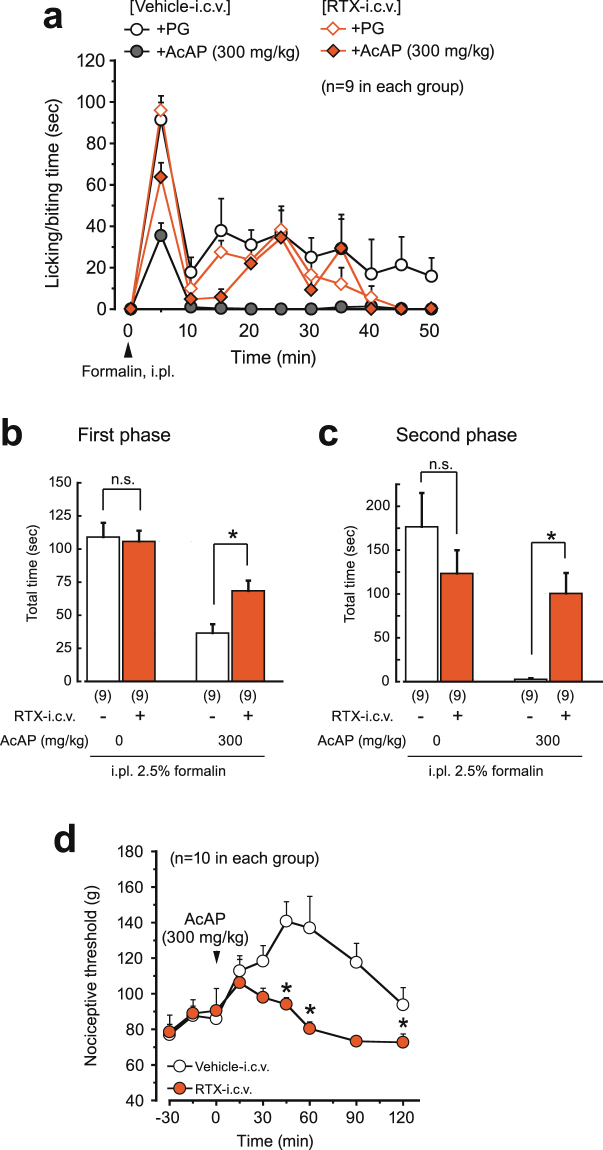
Analgesic effect of acetaminophen in the formalin test and the tail pressure test in RTX-i.c.v.-pretreated mice. Mice were pretreated with RTX (100 ng, i.c.v.) or vehicle one week before the tests. (**a**) Time course of licking/biting behavior evoked by i.pl. injection of 2.5% formalin (arrowhead). Acetaminophen was i.p.-administered 20 min before formalin injection. (**b**, **c**) The first and second phases recorded during 0-10 and 10-50 min after injection of formalin, respectively. (**d**) Change in the threshold for acute mechanical nociception. Acetaminophen was i.p.-administered at time zero (arrowhead). All data represent the mean ± SEM. The number of mice in each group is shown in parentheses. The Mann-Whitney U-test was used for **b**-**d** (*P < 0.05 vs. Vehicle-i.c.v. group). AcAP, acetaminophen; PG, propyleneglycol; n.s., not significant; i.pl., intraplantar injection.

We then examined the analgesic effect of acetaminophen in the RTX test using RTX-i.c.v.-treated mice. In vehicle-pretreated mice, acetaminophen and 4-aminophenol hydrochloride (300 mg/kg, i.p.) reduced the licking/biting behavior, as was the case in naive mice, as described above. However, neither acetaminophen nor 4-aminophenol inhibited the nociceptive behavior in the RTX-i.c.v.-treated mice (Fig. 4c,d,g,h). Also in the formalin test, the licking/biting time of the RTX-pretreated mice was not changed significantly in either the first or second phases. Acetaminophen reduced the time in vehicle-pretreated mice, but was ineffective in the RTX-pretreated mice (Fig. 5a-c). Also in the tail pressure test, the nociceptive threshold of RTX-pretreated mice was not changed, but the analgesic effect of acetaminophen was attenuated (Fig. 5d).

## Discussion

In the present study, we examined nociceptive behavior and the analgesic effect of acetaminophen in the RTX-i.c.v.-treated mice and found that: *1)* intraplantar injection of RTX to the hindpaw evoked TRPV1-dependent nociceptive behavior most effectively at a dose of 1 ng; *2)* RTX-i.c.v.-pretreated mice responded normally to intraplantar injection of RTX, H_2_O_2_ and formalin and application of tail pressure; *3)* acetaminophen reduced the nociceptive behavior evoked by intraplantar RTX; *4)* the analgesic effect of acetaminophen was inhibited in RTX-i.c.v.-pretreated mice. Each of these findings is discussed below.

Intraplantar injection of TRPV1 agonists has been used to assess the function of peripheral TRPV1, and it has been reported that TRPV1-KO mice do not respond to intraplantar capsaicin and RTX^5^. In this study, we initially validated the RTX-evoked pain effect in naive mice and confirmed that the time course and dose-response relationship of the nociceptive behavior were similar to those of the capsaicin test^31^. As with the capsaicin test, a bell-shaped dose-response relationship was also observed in the RTX test. The most effective dose of intraplantar RTX was 1 ng. This dose seems to be reasonable considering that RTX is 500-1000 times more potent than capsaicin *in vivo* ^32^ and that the most effective dose in the capsaicin test was 1600 ng^31^. The RTX-evoked nociception was inhibited by co-administration of capsazepine in addition to s.c. and i.t. pretreatment with RTX. Pretreatment with s.c. and i.t. has been reported to denervate TRPV1-positive primary afferents^10,33^. Our results, therefore, indicate that intraplantar RTX evokes nociception through direct activation of local TRPV1 and that this method can also be used to test the function of peripheral TRPV1.

The striking difference between RTX-i.c.v.-pretreated mice and conventional s.c.- and i.t.-pretreated mice is that i.c.v.-pretreated mice can respond to intraplantar injection of RTX. TRPA1 is co-expressed with TRPV1 in primary afferents^22,23^. Mice treated by the i.c.v. route were also sensitive to TRPA1-dependent, H_2_O_2_-evoked pain, whereas s.c.-pretreated mice were not. These results indicate that TRPV1-positive afferents at the hindpaw are intact in RTX-i.c.v. mice. The RTX-i.c.v. mice also showed a normal response in the formalin test and the tail pressure test. It has been suggested that TRPV1-expressing primary afferents do not contribute to nociception in response to 2.5% formalin^33^. As for mechanical nociception, it has been reported that TRPV1-KO mice respond normally to tail pressure^5^ and that subcutaneous RTX pretreatment and intrathecal capsaicin treatment do not affect the nociception in the paw pressure test^34^ and von Frey test^16^, respectively. On the other hand, it has also been reported that inhibition of nociceptors by combined application of capsaicin and QX-314 increased the nociceptive threshold to paw pinching and von Frey filaments^4^. Although the contribution of TRPV1-expressing afferents to mechanical nociception is uncertain, our results suggest that the sensitivity of RTX-i.c.v. mice to multiple noxious stimuli is normally preserved. However, it should be noted that this finding is not applicable to the orofacial region, because mice treated i.c.v. with RTX lacked the eye-wiping response to capsaicin. The lack of this response suggests that i.c.v.-injected RTX reached the trigeminal nuclei in the pons and the medulla. As mentioned in the Introduction, the extent of desensitization depends on the dose of agonist applied. This technical limitation could be overcome by further optimization of the agonist dose and the time after i.c.v. treatment.

Acetaminophen is the most widely used analgesic drug for several types of pain. Although it has been shown that several agents (e.g. NMDA receptor blockers and tachykinin antagonists) are effective in the capsaicin test^31,35^, the effectiveness of acetaminophen has not been demonstrated. In the present study we demonstrated that acetaminophen and 4-aminophenol alleviated RTX-evoked pain, indicating that acetaminophen is effective for TRPV1-triggered pain. On the other hand, indomethacin and diclofenac, which are widely-used anti-inflammatory drugs, were ineffective in the RTX test. These results indicate that acetaminophen mediates other mechanisms that are independent of cyclooxygenase inhibition^36–38^.

Acetaminophen has been suggested to activate the descending serotonergic system: serotonergic antagonists inhibit the antinociceptive effect of acetaminophen in rodents^39–41^ and humans^42^, and depletion of serotonin inhibits the analgesic effect of acetaminophen in mice^20,40^. In the present study, the level of serotonin in the brain of mice i.c.v.-administered RTX was unchanged. This result is consistent with the previous finding that i.c.v. treatment with capsaicin does not reduce the number of fibers in the medulla that are positive for substance P, and which have also been suggested to contain serotonin^13^. Therefore, the loss of acetaminophen-induced analgesia could not be due to removal of serotonergic neurons in the descending bulbo-spinal system. Acetaminophen has also been suggested to activate central TRPV1 through its metabolic conversion to N-(4-hydroxyphenyl) arachidonamide (AM404) via 4-aminophenol^43^. It has also been shown that both i.p. and i.c.v. administration of capsazepine attenuate acetaminophen-induced analgesia in the formalin test and paw pressure test^21,44^. The results of the present study provide additional evidence for the activation of supraspinal TRPV1 by acetaminophen. Moreover, RTX-i.c.v. mice have an advantage over conventional TRPV1-KO mice in possessing functional TRPV1 primary afferents. This advantage permits distinction between the function of the central and peripheral TRPV1. In the present study, acetaminophen-induced analgesia in the RTX test was lost in RTX-i.c.v. mice, suggesting that acetaminophen mediates the central TRPV1 to exert an analgesic effect on peripheral TRPV1-triggered pain.

The supraspinal regions responsive to loss of the analgesic effect of acetaminophen have not yet been clarified. Because serotonin is not reduced and desensitization at the cervical spinal cord is limited in RTX-i.c.v.-pretreated mice, the target of i.c.v.-injected RTX in the present study would have been at a higher level than the raphe nuclei. Although the precise expression pattern of TRPV1 in the brain has been uncertain, the presence of TRPV1 in the periaqueductal gray (PAG), a primary region involved in the modulation of the descending pain control system, has been reported by some groups^2,45,46^. It has also been demonstrated that TRPV1 activation in the PAG induces analgesia^8^. Moreover, the PAG is a critical region for the analgesic effect of opiates, and it has been shown that systemic treatment with RTX or capsaicin removes TRPV1-positive neurons from the PAG^7^ and inhibits the analgesic effect of morphine^12^. Along these lines, we think that the PAG is the most likely target. The PAG receives inputs from many brain regions, including the hypothalamus^47^ and the amygdala^48^, and TRPV1 expression in these regions has also been reported^45^. Therefore these regions might be targets of i.c.v.-injected RTX.

In summary, we have shown that i.c.v. administration of RTX attenuates acetaminophen-induced analgesia while preserving peripheral TRPV1 function. Based on the preceding findings that central TRPV1 channels are critical for the analgesic effect of acetaminophen, the most probable explanation for the results of the present study is that i.c.v. RTX treatment can induce supraspinal-selective TRPV1 desensitization. This has both clinical and basic implications. In a clinical context, chemical ablation of nociceptive primary afferents is now a promising molecular neurosurgical approach for pain management^49^. Our present results indicate that if the ablation is extended to the brain, the effect of analgesia might be attenuated. Ablation treatments should therefore be performed with care to prevent leakage into the central nervous system. In the context of basic physiology, it has been suggested that TRPV1 also plays some roles in brain function (including nociception, thermoregulation, memory, fear, anxiety, ADHD, and epilepsy), although no brain-specific TRPV1-deficient mutants have yet been generated. The present RTX-i.c.v. mouse model may have potential for investigating the physiological function of supraspinal TRPV1.

## Materials and Methods

### Animals

Male ddY mice (SLC, Shizuoka, Japan) were kept for at least 7 days under a 12-h light/dark cycle before experiments with water and food ad libitum. Five or six week-old mice were used for experiments. All of the experimental protocols used here were approved by the Animal Care and Use Committee of Musashino University. All experiments were conducted in accordance with the guidelines of the Japanese Pharmacological Society.

### Pretreatment with resiniferatoxin

One week before experiments, mice were pretreated with RTX under anesthesia with pentobarbital sodium (60 mg/kg, i.p.; Tokyo Chemical Industry, Tokyo, Japan). For s.c. injection, RTX (0.2 mg/kg) was injected into the back of the neck. For i.c.v. injection, RTX (100 ng) was injected into the right lateral ventricle in a volume of 5 μL via a disposable 27-gauge needle^50^. For i.t. injection, RTX (100 ng) was injected into the subarachnoid space through the intervertebral foramen between L5 and L6 in a volume of 5 μL via a disposable 30-gauge needle according to the method described previously^51^.

### Nociceptive behavior evoked by intraplantar resiniferatoxin, hydrogen peroxide and formalin

Mice were placed individually in a plexiglas cage (29.5 cm × 17.5 cm × 13.5 cm height) and allowed to acclimate to their environment for 30 min. Twenty microliters of RTX, 0.3% hydrogen peroxide or 2.5% formalin was then injected subcutaneously into the plantar surface of the right hindpaw using a 30-gauge needle. In some experiments, 10 μL of RTX was injected into the right forepaw. The incidence of nociceptive behavior characterized by licking/biting of the affected paw was measured in each 5-min block. Either acetaminophen or vehicle (20% propyleneglycol) was administered intraperitoneally 20 min before the intraplantar injection. In the formalin test, the first and second phases were recorded during 0-10 and 10-50 min after injection of formalin, respectively.

### Eye-wiping behavior evoked by corneal application of capsaicin

Mice were placed individually in a plexiglas cage and allowed to acclimate to their environment for 30 min. Ten microliters of 0.1% capsaicin solution was instilled onto the right eye, and the number of eye-wiping episodes was counted for 3 min.

### Tail pressure test

Mice were placed individually in a plexiglas cage and allowed to acclimate to their environment for 30 min. In the tail pressure test, an Analgesy-Meter (Type 7200; Ugo-basile, Varese, Italy) was used to assess the threshold for acute mechanical nociception. Pressure was applied about 1.5 and 2.5 cm from the base of the tail via a blunt probe every 15 min. The pressure (g) required to elicit a response was determined for each mouse and this pressure was defined as the nociceptive threshold. The mean of the two values was used for calculations. The cutoff pressure was 250 g.

### Drugs

Drugs used were resiniferatoxin (LKT Laboratories, St. Paul, MN), acetaminophen (Iwaki seiyaku, Tokyo, Japan), capsaicin and capsazepine (Wako, Osaka, Japan). 4-Aminophenol hydrochloride was obtained from Sigma (St Louis, MO). RTX and capsazepine were dissolved in a mixture of 10% ethanol and 10% polyoxyethylene (20) sorbitan monooleate in physiological saline. For i.c.v. and i.t. injection of RTX, artificial cerebrospinal fluid (ACSF) was substituted for saline. Capsaicin was prepared as a 0.6% stock solution in a mixture of 10% ethanol and 10% polyoxyethylene (20) sorbitan monooleate in physiological saline and later diluted to 0.1% in saline. Acetaminophen was dissolved in 20% propyleneglycol solution. 4-Aminophenol hydrochloride was dissolved in saline just before use to prevent oxidative degradation. For i.p. injection, drugs were injected at 0.1 ml/10 g body weight. RTX was injected in a volume of 5 μL/mouse for i.t. and i.c.v. injection.

### Statistical analysis

All data were expressed as mean ± SEM. The two-tailed Mann-Whitney U-test was used to compare the data for two groups. Two-tailed non-parametric multiple comparisons with Bonferroni correction following the Kruskal-Wallis test were used for multiple comparisons of control and treated groups. Differences at *P* < 0.05 were considered to be significant.

### Data availability

The datasets generated during and/or analysed during the current study are available from the corresponding author on reasonable request.

## Electronic supplementary material

Supplementary information
