# Supplementary material for: Supraspinal-selective TRPV1 desensitization induced by intracerebroventricular treatment with resiniferatoxin
Source: Sci Rep. 2017 Sep 29;7:12452. doi: 10.1038/s41598-017-12717-5 (PMC5622082; doi:10.1038/s41598-017-12717-5)
Supplement: Supplementary file 1 — Supplementary information [file 41598_2017_12717_MOESM1_ESM.pdf]

Supplementary information

Supraspinal-selective TRPV1 desensitization induced by intracerebroventricular treatment with resiniferatoxin

Akihiro Fukushima[1,2], Kizuku Mamada[1], Aki Iimura[1] and Hideki Ono[1,2] *

1 Laboratory of Clinical Pharmacy and Pharmacology, Faculty of Pharmacy, Musashino University, 1-1-20 Shinmachi, Nishitokyo-shi, Tokyo 202-8585, Japan

2 Research Institute of Pharmaceutical Sciences, Musashino University, 1-1-20 Shinmachi, Nishitokyo-shi, Tokyo 202-8585, Japan

Supplemental Methods

Spontaneous locomotor activity in a novel environment

On the experimental day, mice were transferred to a room lit by a red dim light (70 lux) and allowed to acclimate to their environment for 60 min. The mice were then individually placed in a novel cage and subsequent spontaneous locomotor activity was measured using an ACTIMO-100 Infrared Beam Sensor System (Bio Research Center, Nagoya, Japan). Total activity counts in each 5-min block were automatically recorded for 60 min.

LC-MS/MS analysis of monoamines in the mouse brain

Monoamine contents in mouse brain were analyzed by liquid chromatography coupled with tandem mass spectrometry (LC-MS/MS) according to the method described previously[1]. Briefly, mouse brains were homogenized in solution containing 0.05% Triton-X and 10 mM ammonium formate and centrifuged with ice-cold chloroform/methanol (2:1, v/v). 3,4-Dihydroxybenzylamine hydrobromide (Sigma, St Louis, MO) as an internal standard was added to the homogenate. Serotonin, noradrenaline and dopamine were extracted from the supernatant with a Monospin PBA column (GL Sciences, Tokyo, Japan). Samples were analyzed on a Shimadzu LC-MS/MS system (LCMS-8040; Kyoto, Japan) equipped with a COSMOSIL PBr column: 2.0 mm I.D. × 150 mm (Nakalai Tesque, Kyoto, Japan). All samples were tested in duplicate.

Supplemental Reference

1. Fukushima, A., Sekiguchi, W., Mamada, K., Tohma, Y. & Ono, H. Serotonergic System Does Not Contribute to the Hypothermic Action of Acetaminophen. *Biol. Pharm. Bull.* **40,** 227–233 (2017).

Supplemental Figure legends

Figure S1. Mice i.c.v.-administered RTX show normal spontaneous activity and brain monoamine content. (**a**) Spontaneous locomotor activity in a novel cage. Two-way repeated measures ANOVA demonstrated a significant main effect of time [F(11,198) = 70.888, $P<0.05$]. However, there was no significant main effect of treatment [F(1,18) = 0.140, $P>0.05$] or any interaction between these factors [F(11,198) = 1.545, $P>0.05$]. (**b-d**) Brain content of serotonin (**b**), noradrenaline (**c**) and dopamine (**d**). All data represent the mean (± SEM) from ten (**a**) and seven mice (**b**). The Mann-Whitney U-test was used for (**b-d**) ($P\geq0.05$).

Figure S2. Ineffectiveness of non-steroidal anti-inflammatory drugs in the RTX test. Time spent licking/biting in response to i.pl. injection of RTX (1 ng) during the first 10 minutes of the RTX test. Indomethacin (**a**) and diclofenac sodium salt (**b**) were i.p.-administered 20 minutes before the test. All data represent the mean ± SEM. The number of mice in each group is shown in parentheses. The Mann-Whitney U-test was used ($P\geq0.05$).

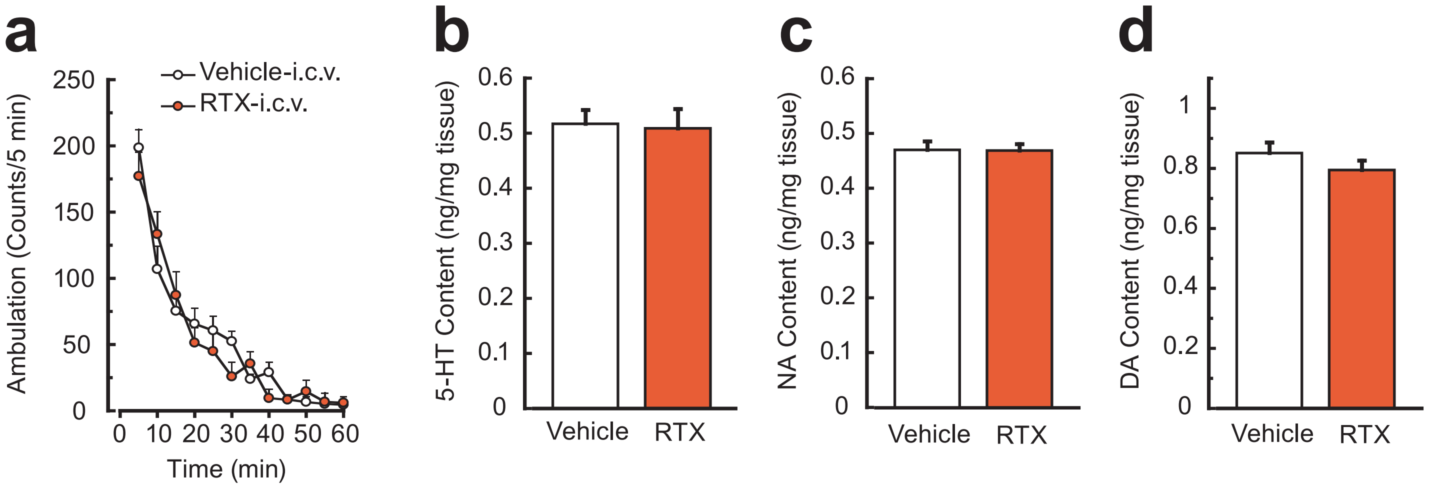
a
Vehicle-i.c.v.
RTX-i.c.v.
Ambulation (Counts/5 min)
250
200
150
100
50
0
0 10 20 30 40 50 60
Time (min)
b
5-HT Content (ng/mg tissue)
0.6
0.5
0.4
0.3
0.2
0.1
0
Vehicle
RTX
c
NA Content (ng/mg tissue)
0.6
0.5
0.4
0.3
0.2
0.1
0
Vehicle
RTX
d
DA Content (ng/mg tissue)
1
0.8
0.6
0.4
0.2
0
Vehicle
RTX

Figure S1

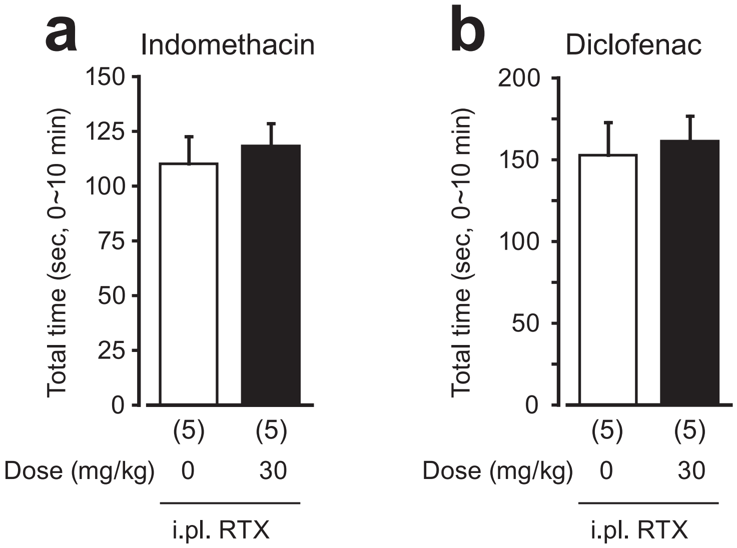

a
Indomethacin
150
125
100
75
50
25
0
Total time (sec, 0~10 min)
(5)
(5)
Dose (mg/kg)
0
30
i.pl. RTX
b
Diclofenac
200
150
100
50
0
Total time (sec, 0~10 min)
(5)
(5)
Dose (mg/kg)
0
30
i.pl. RTX

Figure S2
